# Effects of the ECHO tele-mentoring program on HIV/TB service delivery in health facilities in Zambia: a mixed-methods, retrospective program evaluation

**DOI:** 10.1186/s12960-023-00806-8

**Published:** 2023-03-20

**Authors:** Brian Mubanga, Sombo Fwoloshi, Lastina Lwatula, Nomsa Siamwanza, Khozya Zyambo, Henry Sichinga, Hannah Tappis, Lloyd B. Mulenga, Aurthur Moonga, Lunga Ziko, Faith Simushi, Harry Madimba Massamba, Given Hapunda, Francis Sichimba, Hellen Mtonga, Maybin Kalubula

**Affiliations:** 1 Zambian Ministry of Health, Ndeke House, Haile Selassie Avenue, Lusaka, Zambia; 2 Jhpiego Zambia Country Office, 8 Ngumbo Road, Longacres, Lusaka, Zambia; 3 Jhpiego, 1615 Thames Street, Baltimore, MD 21231 USA

**Keywords:** ECHO, HW21, HIV/TB, HIV/AIDS, HIV/TB ECHO, HRH, Telementoring

## Abstract

**Background:**

In the quest to ensure that quality healthcare is provided to all citizens through building healthcare worker capacity and extending reach for expert services, Zambia’s Ministry of Health (MoH) in collaboration with its partners PEPFAR through the CDC and HRSA, began to implement the Extension for Community Healthcare Outcomes (ECHO) tele-mentoring program across the country through the Health Workers for the 21st Century (HW21) Project and University Teaching Hospital HIV/AIDS Project (UTH-HAP). This ECHO tele-mentoring approach was deemed pivotal in helping to improve the human immunodeficiency virus (HIV) service delivery capacity of health care workers.

**Method:**

The study used a mixed method, retrospective program evaluation to examine ECHO participants’ performance in the management of HIV/AIDS patients in all the 10 provinces of Zambia.

**Case presentation:**

A phenomenological design was applied in order to elicit common experiences of ECHO users through focus group discussions using semi-structured facilitation guides in four provinces (Eastern, Lusaka, Southern and Western) implementing ECHO tele-mentoring approach. These provinces were purposively selected for this study. From which, only participants that had a monthly frequency of ECHO attendance of ten (10) and above were selected. The participants were purposively selected based on the type of cadre as well as facility type so that the final sample consisted of Doctors, Nurses, Midwives, Clinical Officers, Medical Licentiates, Pharmacy and Laboratory Personnel. All sessions were audio recorded and transcribed by the data collectors. A thematic content analysis approach was adopted for analyzing content of the interview's transcripts.

**Results:**

Enhanced knowledge and skills of participants on HIV/TB improved by 46/70 (65.7%) in all provinces, while 47/70 (67.1%) of the participants reported that ECHO improved their clinical practice. Further, 12/70 (17.1%) of participants in all provinces reported that presenter/presentation characteristics facilitated ECHO implementation and participation. While, 15/70(21.4%) of the participants reported that ownership of the program had contributed to ECHO implementation and participation. Coordination, another enabler accounted for 14/70 (20%). Inclusiveness was reported as a barrier by 16/70 (22.8%) of the participants while 6/70 (8.6%) of them reported attitudes as a barrier (8.6%) to ECHO participation. In addition, 34/70 (48.6%) reported poor connectivity as a barrier to ECHO implementation and participation while 8/70 (11.5%) of the participants reported that the lack of ownership of the ECHO program was a barrier. 22/70 (31.4%) reported that increased workload was also a barrier to the program’s implementation.

**Conclusion:**

Consistent with its logical pathway model, healthcare providers’ participation in ECHO sessions and onsite mentorship contributed to improved knowledge on HIV/TB among health care providers and patient health outcomes. In addition, barriers to ECHO implementation were intrinsic to the program its self, such as coordination, presenter and presentation characteristics other barriers were extrinsic to the program such as poor connectivity, poor infrastructure in health facilities and negative attitudes towards ECHO. Improving on intrinsic factors and mitigating extrinsic factors may help improve ECHO outcomes and scale-up plans.

## Introduction

The Zambia MOH envisioned the tele-mentoring Extension for Community Healthcare Outcomes (teleECHO©) model developed by Project ECHO of the University of New Mexico (UNM) as an efficient way to strengthen and expand the capacity of health care providers to deliver quality health services to ensure improved health outcomes [1]. Application of the teleECHO model deploys and uses technology to foster linkages between mid and lower level health care providers at health facilities to subject matter experts and specialists based at centers of excellence or local academic medical centers [2, 3]. In addition, the teleECHO model provides a platform for the low-dose high-frequency (LDHF) training approach, which emphasizes delivery of need-based knowledge in appropriate doses on a regular basis [3, 4]. The LDHF approach minimizes health care providers' time away from health facilities, thereby improving retention and quality of care [4].

As in many low- and middle-income countries, a shortage of trained health professionals, and gaps in health workforce capacity to manage complex disease conditions are barriers to controlling the HIV epidemic in Zambia [5]. According to the Zambia Population-based Health Impact Assessment (ZAMPHIA) of 2016, the HIV prevalence was at 11.2% with women being disproportionately affected with 14.6% [7]. According to national statistics, 48,000 new infections per year were noted among adolescents and young women. According to the UNAIDS report of 2018, 1.2 million people were on ART (antiretroviral therapy) in Zambia while 67% were reported to have been virally suppressed. The biggest cause of mortality and morbidity among PLHIV (people living with HIV) in Zambia is tuberculosis (TB), and studies across the world have shown that TB treatment preventive treatment (TPT) can reduce mortality by 37%, but this figure is only achieved if this treatment is completed [8].

The MOH and collaborating partners have implemented ECHO activities in Zambia to address key human resources for health (HRH)-related barriers to achieving UNAIDS’ 95-95-95 targets [5], including workforce development and HRH system gaps [5, 6]. The ECHO model was selected as a strategy due to its catalytic effect in demonopolization of knowledge to enhance connections between HIV clinical specialist/subject matter experts and networks or communities of practice [2, 3].

Studies in other countries have shown that this approach helps strengthen and improve health care provider’s ability to provide best practices in HIV care and treatment for improved health outcomes [1–3, 9]. However, studies in other countries have also shown that effectiveness of the approach in improving health worker knowledge and confidence; few examine effects on provider practices or patient outcomes. In fact, to the best of our knowledge, only one evaluation has examined the effects of the ECHO approach in improving provision of HIV services in sub-Saharan Africa. The study, evaluating ECHO implementation at 10 clinical sites in Namibia between 2015 and 2016, found that the approach improved health worker knowledge and satisfaction, and decreased participants’ feelings of professional isolation [12]. Since then, ECHO implementation has expanded rapidly. Prior to the start of the COVID-19 pandemic, there were more than 30 Project ECHO programs related to HIV/TB being implemented in 14 countries in sub-Saharan Africa, along with several multi-country Project ECHO programs that engage more than 40 countries across sub-Saharan Africa [12].

This evaluation, conducted after 18 months of the Zambia HIV ECHO program implementation, was therefore designed to contribute to global learning by examining effects of the intervention on facility-based HIV service delivery, and identifying factors that enabled and hindered implementation.

## Methodology

### Study design

This was a mixed-methods, retrospective program evaluation study, conducted amidst the first wave of the COVID-19 pandemic in Zambia, in August 2020. Multiple primary and secondary data sources were used to address evaluation questions. First, process and performance indicators were extracted from routine program monitoring and reporting systems (Project ECHO’s iECHO system and PEPFAR’s Data for Accountability, Transparency and Impact Monitoring [DATIM] system]. Then, a facility record review was conducted to follow up on outcomes of cases presented during ECHO sessions. Health workers and managers were also involved in the evaluation to complete an online survey and participate in focus group discussions.

### Intervention description and study setting

The ECHO model uses videoconference technology to create virtual communities of practice that allow providers to care for patients with complicated health problems, which they would otherwise refer to a higher level of care. The model virtually links a specialist expert team at a “hub” with outlying “spokes”, which are providers at health facilities. Together, they make up the network, a community of practice that participates in weekly virtual ECHO sessions. During these sessions, health workers from the spoke sites present real-world patient case scenarios (de-identified) or health system case studies they have difficulty managing and discuss options with other providers and experts in the network. Brief didactic presentations are made to enhance participants’ knowledge on specific subjects. During an ECHO session, participants learn from one another and experts learn from their learners thus ‘Teach All, Learn All’. The continuous loop of learning, mentoring and peer support is what makes ECHO unique and different from a webinar, e-learning course or telemedicine. The ECHO model brings together experts from multiple focus areas for a holistic approach. This model has a multiplier effect, through ECHO participation, a single expert contributes to the development of expertise in 20 or more health care providers who in turn utilize the expertise knowledge and skills in their communities to improve the quality of lives of people living with HIV and other related conditions. Following an ECHO session, the ECHO facilitator consolidates all the pharmacological and non-pharmacological solutions and provides written recommendations. These ECHO recommendations are also documented in the patient case follow-up form.

The Zambia MOH HIV ECHO program was launched in October 2018 with one hub and six spoke health facilities, and expanded over the next 12 months to 64 spokes across Eastern, Lusaka, Western, and Southern provinces by October 2019.

Implementation is led by the MOH with support from the HW21 project, a consortium of partners led by Jhpiego funded by Health Resources and Services Administration (HRSA); alongside UTH-HAP and the mentorship program that is funded by the U.S. Centers for Disease Control and Prevention (CDC) [10].

### Sampling, data collection and analysis

#### Sampling

1. **Health worker survey**
  The study surveyed 88 health workers. The sample was determined by using the iECHO database to determine the potential number of health workers who will meet the participation inclusion criteria (health workers who have attended at least one ECHO session per month for at least 12 months) by March 2020. These are health workers who had attended at least one session per month for 8 months by November 2019.
2. **Focus group discussions (FGDs)**
  Eight (8) focus group discussions were conducted with 12 participants in each, resulting in 96 participants spread across 4 provinces. Each province conducted two categories of FGDs; one for managerial level staff comprising a mix of health facility managers (4), ECHO session facilitators/coordinators (4) and mentors (4); the other category comprised strictly of health workers representing different cadres in the same proportion as the population of ECHO attendees; Doctors (2), Nurses (6), Clinical Officers (1), Medical Licentiates (1), Pharmacy Personnel (1) and Laboratory Personnel (1). Below are further details of the sampling process (Table 1).

**Table 1:** Sample size of health facilities and study participants

Data collection method	Study participants in each province				Total sample
	Eastern	Western	Lusaka	Southern	
*Study participants*					
Health worker survey	19	23	30	16	88
Focus group discussions for health workers—one group per province	12	12	12	12	48
Focus group discussions for managers and facilitators—one focus group overall	12	12	12	12	48

### Key performance indicator analysis

The study used a difference-in-differences (DiD) quasi-experimental design to measure the effect of implementing the ECHO program on key outcomes at health facilities in the Eastern, Lusaka, Southern and Western provinces of Zambia. Broadly, the DiD approach evaluates the difference between the changes in outcomes over time for an intervention group (i.e., facilities that implemented the ECHO program) and a control group (i.e., facilities that did not implement the ECHO program). By assuming that the trend in outcomes would have been the same across both groups in the absence of the intervention, DiD enables us to estimate the effect of the intervention.

Aggregate data for all facilities in the four provinces were abstracted from DATIM (Data for Accountability Transparency and Impact Monitoring) for the periods July–September 2018 (pre-intervention) and October 2018–March 2020 (intervention). All health facilities in the four provinces that reported nonzero results for the selected outcomes during both the pre-intervention and intervention periods were included in the analysis. DATIM is a web-based system used by all PEPFAR implementing partners to report the impact of their programs.

The number of intervention facilities and non-intervention facilities included in the analysis varied by indicator due to variability in services offered and reported at each facility. The inclusion of all health facilities that did not implement ECHO were elected as control group (vs. selecting a matched comparison control group) due to limitations on the information available to identify appropriate matched controls.

DATIM key performance indicators of interest in the analysis were outcome indicators related to HIV testing services (HTS), TB preventative therapy (TPT), and HIV viral load (VL) testing. Key performance indicator definitions are described in Table 2.

**Table 2:** Selected PEPFAR key performance indicators and definitions

Indicators	Definitions
HTS yield	Number of positive HIV tests divided by the total number of individuals who received HIV testing per national guidelines
OPD yield	Number of positive HIV tests conducted in the outpatient department (OPD) divided by the total number of individuals who received HIV testing per national guidelines in the OPD
Index testing yield	Number of index testing contacts who were identified HIV-positive divided by the total number of index contacts who received HTS and received their test results. Index testing contacts were defined as sexual contacts or biological children of PLHIV who were elicited and offered HTS
TPT completions	Number of ART patients who completed a course of TB preventive therapy during the reporting period [for continuous IPT programs, this includes the patients who have completed the first 6 months of isoniazid preventive therapy (IPT)]
Expected TPT completions	Number of ART patients who are expected to complete a course of TB preventive therapy during the reporting period (for programs using continuous IPT, this includes only the patients who are scheduled to complete the first 6 months of therapy)
Viral load coverage	Number of PLHIV with documented HIV viral load test results during the last 12 months divided by the number of PLHIV eligible for viral load testing
Viral load suppression	Number of PLHIV with viral load test results < 1000 cp/ml during the previous 12 months divided by the number with any HIV viral load test result

Models included terms for intervention (ECHO vs. non-ECHO), period (pre-intervention vs. post-intervention), and an interaction between the two variables which represented differences in outcomes pre- vs. post-intervention between ECHO and non-ECHO sites. The interaction term, which is referred to as the DiD coefficient, and was the primary measure of interest. Outcome variables were log-transformed when warranted by model assumptions and all models included covariates for the pre-intervention number of PLHIV on treatment at a given health facility and the province where the health facility was located.

The analysis period for DiD was predefined by the study team at the onset of the study. Outcomes were assessed using linear mixed-effects models with random intercepts for participants’ repeated measures over time. The pre-intervention period was defined as July 2018–September 2018 and the post-intervention period was either October 2019–December 2019 or January 2020–March 2020 (depending on the outcome being analyzed). Analyses for DiD were conducted with the aid of the computer software R v4.02 (R foundation for statistical computing, Vienna, Austria) using the nonlinear mixed-effects package, while extracted data for case records were analyzed using SPSS v22.

### Health facility record review

To explore HIV care practices among healthcare workers in Zambia, the study conducted a health facility record review to assess health worker adherence to case-specific guidance provided during ECHO sessions, here forth referred to as “ECHO recommendations”.

The data collection was conducted by independent consultants and Ministry of Health Personnel. Teams visited all facilities with HIV and TB cases presented during ECHO sessions facilitated between October 2018 and March 2020. At each facility, teams requested access to these case files and used a standardized data collection form to extract information on healthcare worker adherence to recommended treatment protocols discussed during ECHO sessions and resulting patient outcomes. Data editing and cleaning included structure and consistency checks to ensure completeness and validity.

### Focus group discussions with health workers and managers

Focus group discussions were conducted to explore experiences of health workers and managers involved in the ECHO tele-mentoring program in each province. Participants of these discussions were selected based on their participation in HIV/TB ECHO sessions. To avoid potential selection bias, the consultants purposively selected using a prescribed criterion as highlighted in the sampling.

Data collection and analysis was conducted by two independent consultants, both experienced in collecting qualitative data. HW21 and MOH staff oriented the consultants on the study design, methods, and ethics prior to the initiation of fieldwork. After the familiarization, the consultants participated in the field-testing of data collection instruments in Lusaka. Data collection was undertaken from August 6 to August 15, 2020.

Participants were purposively selected to include a mix of cadres (nurses, clinical officers, medical doctors, hospital managers), facility types, and levels of participation (attendance) in ECHO sessions. In each province, up to 12 health workers (a mix of nurses, lab technicians, pharmacists, clinical officers, and medical doctors) and 12 managers (a mix of health facility in-charges, mentors, ECHO coordinators, and other facility managers) involved in the program were invited to participate in separate focus group discussions in the provincial capital.

The discussions were conducted using semi-structured facilitation guides designed to elicit open reflection and discussion of experiences participants had in the program. All sessions were audio recorded and transcribed by the data collectors. A phenomenological lens and thematic content analysis approach was adopted for analyzing content of the interview's transcripts. Both manifest and latent analysis were conducted. Data were first independently coded after which the consultants met to discuss areas of agreement and disagreements. Areas of disagreement were discussed until commonly agreed codes were identified. This process was met to ensure trustworthiness of the analysis.

Thereafter, the consultants developed a codebook with deductive codes that aligned with the study objectives. Thematic analysis was aided by using an iterative process of reviewing, comparing, and contrasting the data to identify emergent patterns at impact, knowledge, practice level, as well as factors enabling and hindering ECHO implementation in each province. This iterative process allowed for a rigorous probing of the data to develop categories, identify themes, and map insights into factors enabling and hindering implementation of the ECHO approach.

### Triangulation and synthesis

Preliminary findings for each component of the evaluation were shared with HW21, UTH, MOH, and consultant study team members at a series of virtual workshops in September and October 2020. Methodological strengths and limitations, key findings, and value of insights from each component were discussed and taken into consideration in finalizing analyses. Study team members then reviewed findings from all evaluation components, reflecting on implications for ongoing and future implementation of the ECHO approach in Zambia.

## Ethical considerations

The study was reviewed and approved by the ERES Converge Institutional Review Board and National Health Research Authority in Lusaka, Zambia with reference number IRB00011734. In addition, the Johns Hopkins Bloomberg School of Public Health Institutional Review Board (IRB) in Baltimore, Maryland, USA (United States of America), reviewed the evaluation plan and determined that it was not human subjects research and therefore did not require IRB oversight (IRB No. 11734).

## Results

### Intervention characteristics and participation by province

A total of 64 sites participated in ECHO sessions between October 2018 and March 2020, with 3258 health workers registering for at least one ECHO session. Lusaka and Southern provinces recorded the highest participation of health workers in ECHO sessions. Each ECHO session had an average attendance of 148 health workers. During this period, an average of 111 health workers participated in at least two ECHO sessions per month, with the highest number being 256 recorded in February 2020, while the lowest was 13 recorded in January 2019.

Sixty-four HIV/TB-specific didactics were presented at ECHO sessions during the first 18 months of program implementation, 57 of these included HIV/TB-specific cases encountered in the different health facilities while 7 were knowledge updates without cases.

### Effect of intervention on HIV service outcomes

The study employed six (6) key HIV indicators whose outcomes were compared by the presence and absence of the ECHO model at the health facility. Both ECHO and non-ECHO facilities showed an increase in the means of all key HIV indicators over the period under study. Table 3 shows the details.

**Table 3:** Summary of intervention characteristics and participation by province

Characteristics	Eastern Province	Lusaka Province	Southern Province	Western Province	Total
Number of spokes participating in ECHO sessions	13	22	16	13	64
Number of participants ever registered for ECHO program	710	1206	745	597	3258
Average number of registered participants for each session	91	137	76	60	148
Number of regular participants in ECHO sessions	21	44	20	26	111
Number of HIV/TB related cases presented in ECHO sessions	13	19	7	18	57

Table 4 presents statistical results of outcomes of the six key HIV program indicators. For index testing yield and VL coverage, results indicate that ECHO facilities were associated with absolute increases of 5.37% and 13.24% following ECHO implementation, respectively. However, only the VL coverage increase was statistically significant. ECHO facilities were also associated with statistically significant increase in TPT completions in the post-intervention period (Table 5).

**Table 4:** Mean reported outcomes at intervention and non-intervention facilities pre- and post-intervention

Indicator	Health facility type	*n*	Pre-intervention mean^a^	Post-intervention mean^a^
HTS yield	ECHO	59	5.86 ± 3.46	6.62 ± 3.45
	Non-ECHO	709	3.17 ± 3.29	3.90 ± 3.50
OPD yield	ECHO	59	5.48 ± 3.52	7.10 ± 4.03
	Non-ECHO	672	3.32 ± 3.58	4.24 ± 4.81
Index yield	ECHO	32	16.09 ± 12.85	24.93 ± 15.68
	Non-ECHO	137	17.37 ± 20.26	21.12 ± 20.47
TPT completion rate	ECHO	37	78.18 ± 29.10	81.73 ± 23.57
	Non-ECHO	144	84.24 ± 26.01	78.80 ± 26.14
VL coverage	ECHO	52	42.24 ± 23.07	82.65 ± 11.37
	Non-ECHO	351	41.82 ± 26.11	68.99 ± 19.52
VL suppression	ECHO	55	83.57 ± 12.55	93.27 ± 4.44
	Non-ECHO	412	79.28 ± 16.96	89.96 ± 9.30

^a^Means expressed as percentages ± standard deviation

**Table 5:** DiD coefficients, relative changes, and absolute changes

Outcome	*n*	DiD coefficient (95% CI)^a^	*p* value	Absolute change^b^
HTS yield	1536	− 12.63 (− 32.50–13.09)	0.306	− 0.74%
OPD yield	1462	− 0.024 (− 25.47–27.63)	0.856	− 0.13%
Index yield	338	33.37 (− 20.15–122.78)	0.269	+ 5.37%
TPT completions	362	16.53 (− 11.84–54.03)	0.281	+ 16.53%
VL coverage	806	13.24 (4.11–22.38)	0.0046	+ 13.24%
VL suppression	934	− 0.98 (− 5.35–3.40)	0.661	− 0.98%

^a^Model includes log-transformed outcome variable and DiD coefficient (95% CI) is exponentiated

^b^Absolute change is calculated by multiplying the DiD coefficient by the pre-intervention mean at ECHO sites (for models with log-transformed outcome variables) or is equivalent to the DiD coefficient (for models that do not have log-transformed outcome variables)

### HIV-related practice among health workers in ECHO implementation sites

The total number of ECHO cases were 71 which were presented over a series of 64 ECHO sessions and of these; 57 were HIV/TB related cases and 53 of these cases met the criteria for assessment. The study revealed that 44 (83%) cases had information on provider adherence to ECHO recommendations and 44 (83%) cases had information on health outcomes while 35 (79%) cases recorded positive health outcomes. There were 9 (17%) cases with negative health outcomes (6 died and 3 alive but with deteriorating health).

## Factors enabling and hindering ECHO implementation

### Impact of intervention on HIV service outcomes

Positive trends were evident across ECHO and non-ECHO health facilities in our descriptive assessment of pre- vs. post-intervention means for all outcomes (Table 6).

**Table 6:** Descriptive assessment of pre- and post-intervention means of outcomes

Indicator	Health facility type	*n*	Pre-intervention mean	Post-intervention means
HTS yield	ECHO	59	5.86%	6.62%
	Non-ECHO	709	3.17%	3.90%
OPD yield	ECHO	59	5.48%	7.10%
	Non-ECHO	672	3.32%	4.24%
Index yield	ECHO	32	16.09%	24.93%
	Non-ECHO	137	17.37%	21.12%
TPT completions	ECHO	37	114	224
	Non-ECHO	116	46	51
Expected TPT completions	ECHO	38	145	322
	Non-ECHO	136	50	65
VL coverage	ECHO	52	42.24	82.65
	Non-ECHO	351	41.82	68.99
VL suppression	ECHO	55	83.57	93.27
	Non-ECHO	412	79.28	89.96

Table 7 presents the estimates of our primary outcomes of interest. For index testing yield and VL coverage, estimates indicate that ECHO facilities were associated with absolute increases of 5.37% and 13.24% following ECHO implementation, respectively, though the estimate for index testing yield was not significant. ECHO facilities were also associated with an average of 157 additional TPT completions and 162 additional expected TPT completions in the post-intervention period.

**Table 7:** Primary outcomes

Outcome	*n*	DiD coefficient (95% CI)	*p* value	Relative change	Absolute change
HTS yield	1536	− 12.63 (− 32.50–13.09)	**0.306**	− 12.63%	− 0.74%
OPD yield	1462	− 0.024 (− 25.47–27.63)	**0.856**	− 2.44%	− 0.13%
Index yield	338	33.37 (− 20.15–122.78)	**0.269**	+ 33.37%	+ 5.37%
TPT completions	306	138.14 (35.93–316.62)	**0.0026**	+ 138.14%	+ 157
Expected TPT completions	348	111.91 (25.73–257.15)	**0.0050**	+ 111.91	+ 162
VL coverage	806	13.24 (4.11–22.38)	**0.0046**	+ 31.34%	+ 13.24%
VL suppression	934	− 0.98 (− 5.35–3.40)	0.661	− 1.17%	0.98%

^a^Model includes log-transformed outcome variable and DiD coefficient (95% CI) is exponentiated

^b^Relative change is equivalent to the DiD coefficient (for models with log-transformed outcome variables) or is calculated by dividing the DiD coefficient with the pre-intervention mean at ECHO sites (for models that do not have log-transformed outcome variables)

^c^Absolute change is calculated by multiplying the DiD coefficient by the pre-intervention mean at ECHO sites (for models with log-transformed outcome variables) or is equivalent to the DiD coefficient (for models that do not have log-transformed outcome variables)

On the other hand, adherence to ECHO recommendations was mainly limited by lost to follow-up (29.4%) followed by non-availability of the recommended drugs (ARTs) for the cases where drug switch was advised by the subject matter expert. The vast majority of reported reasons for non-adherence to ECHO recommendations were more of health systems rather than client-level factors. Suffice to say, non-adherence was mainly due to circumstances beyond the ECHO site’s control as all sites were willing to implement the recommendations.

Further, the availability of basic laboratory results was a significant challenge in all sites visited as approximately 28% of cases did not have VL results in the medical records at case presentation and about 43.4% did not have creatinine and hemoglobin results at the time of case presentation. The availability of VL, creatinine, and hemoglobin results further declined (39.6%, 20.8%, and 15.1%, respectively) at 3- and 6-month follow-up.

Out of the 53 cases that were assessed, 44 (83%) cases had complete information on health outcomes, 35 (79%) cases recorded positive health outcomes, 9 (17%) cases had negative health outcomes (6 dead and 3 alive but with deteriorating health). Five of the cases with negative health outcomes were from Western Province, two were from Eastern Province. Lusaka and Southern provinces had one case each. Of the 9 negative outcomes, 7 of the cases were male, 2 were female. Non-adherence to recommendations was recorded in five of the cases with negative health outcomes.

## Experiences and perspectives reported by health worker survey participants

Findings showed that none of the health workers surveyed had presented a case for ECHO session. Most participants indicated that patient cases presented by other providers were most useful. Also, the majority of health workers (90%), comprising 21 clinical officers, 27 nurses, 5 laboratory personnel, 4 pharmacy personnel, and 1 doctor reported that the availability of CPD credits encouraged them to participate in ECHO sessions.

The majority of providers (78%) also reported that enhanced knowledge of HIV care and prevention services through technical updates was the biggest benefit of participating in ECHO sessions, followed by having access to experts at UTH and other health facilities (20.6%) as a factor in enhancing knowledge. Only 1.4% providers expressed participating in ECHO sessions as a benefit (Table 7).

Ultimately, the most common barrier (74%) to participation in ECHO sessions was not having the time to attend due to their workload; 7%, respectively, reported a lack of awareness of the ECHO session schedules or no interest in the content provided in ECHO sessions as their barriers to participation (Tables 8, 9, 10).

**Table 8:** Component of the ECHO sessions participants found most useful (*N* = 64)

Component of the ECHO session most useful	*N* (%)
Didactic presentations given during the ECHO session	16 (24%)
Patient cases presented by other providers, and the specialist guidance and discussion associated with those cases	45 (66%)
Patient cases I present during the ECHO sessions	0 (0%)
Sharing knowledge and skills with peers in my health facility based on the information from the ECHO session	7 (10%)

**Table 9:** Perceived benefits from ECHO participation (*N* = 68)

In your opinion, what is the biggest benefit of participating in ECHO sessions?	*N* (%)
Access to experts at UTH and other health facilities	14 (20.6%)
Opportunity to develop professional relationships/discussions with peers	1 (1.4%)
Enhanced knowledge of HIV care and prevention services through technical updates	53 (78%)
Other	0 (0%)

**Table 10:** Biggest barrier to participation in ECHO sessions (*N* = 31)

What are the biggest barriers to your participation in the ECHO sessions?	*N* (%)
No time to attend the sessions due to my workload	23 (74%)
Lack of awareness of the ECHO session schedules	4 (13%)
No interest in the content provided in the ECHO sessions	4 (13%)
Other	0 (0%)

## Experiences and perspectives reported by focus group discussion participants

Focus group discussion participants identified a number of factors affecting implementation of the tele-mentoring intervention, some intrinsic to the ECHO approach and others reflective of variations in leadership support and implementing environments across project sites.

### Enablers intrinsic to ECHO approach

#### Technology

Attributes of the ECHO approach that were seen as key features enabling successful implementation included the choice of technology platform and the content, structure, and delivery of presentations, particularly the emphasis on peer interaction.

First, the Zoom technology platform was described as an effective enabler because it was user friendly in that even those cadres with limited technological skills were able to adapt and use the Zoom technology with few difficulties. Several participants perceived Zoom as economical to use because it was accessible anywhere in the country, even in hard to reach places with limited internet connectivity, and noted that it had grown in popularity and use since the start of the COVID-19 pandemic.

Another important strength of the ECHO program was its wide coverage and accessibility. Participants shared that whether one was at work or at home, access was easy, hence it was able to reach many health workers at the same time. ECHO was facilitated by the ease of access even when one was away from the facility where ECHO equipment was installed.

#### Presenters

The participants felt that participation was facilitated by confidence in presenters especially when presenters were seen to be knowledgeable, confident, organized, and experts in their field, which inspired trustworthiness in what they were teaching. Additionally, participation was inspired by case scenarios, which related to challenges faced by health facilities.

*“One thing that I liked about ECHO is that they introduced a topic, discuss it and then they will do a case study. Then we go through how to handle that case systematically ensuring that all the steps carried out are uniformly done.”–Health worker, Lusaka Province*

Topics were also reported to facilitate participation in ECHO sessions, especially when the topics were of interest (related to work of the health worker) or responded to the challenges that the health worker was facing in terms of patient management. For example, topics like hepatitis B and COVID-19 were found to be more interesting to ECHO participants.

*“HIV/TB services in my district have improved because of ECHO sessions. We now have proper patient management. For example, we had a case of hepatitis B where after the presentation it was clear that no medication was supposed to be given to the patient and that there were stages of follow-up which were supposed to be made for the patient. These were not planned before the ECHO sessions. So, before the ECHO session a lot of drugs were being wasted to give to hepatitis B patients who were not eligible to be given medication.”—Manager, Lusaka Province*

Additionally, the participants noted that sessions were most effective when the presenters were audible and clear, used less jargon, and involved everybody in their facilitation. They also reported that polling questions were important because they enabled them to self-assess what they knew about the subject matter and motivated them to want to know more about the subject. It was also noted that presenters not being dismissive and being respectful helped inspire participation.

#### Peer and expert interaction

Finally, peer interaction was cited as a key enabler for ECHO implementation success. Participants described ECHO sessions as having created an enabling environment that is inclusive but also encouraged participation of all cadres regardless of discipline to contribute equitably, with autonomy and comfort of being heard.

Some participants noted that having access to experts via ECHO sessions gave them confidence to manage difficult cases to consult for help when needed. ECHO linked providers with experts and also enabled health workers to network within and across districts and provinces. These peer networks became important when experts were not available and also for some cadres who reported not feeling comfortable consulting renowned experts:

- Program and context-specific enablers—Program and context-specific enablers identified by focus group discussion participants included perceived personal and health system benefits to participation, facility culture, and communication.

##### Continuous professional development (CPD)

Some participants shared that they were motivated by perceived personal benefits, such as career development. Most participants recognized that the field of HIV/TB was dynamic and fast evolving thus, ECHO sessions provided them with opportunities to update their knowledge and skills.

*“I have never gone for a workshop for third line treatment. It is just the interest, I have just been reading and the ECHO sessions that we were having with professors, they really enlightened us and we are able to manage third line clients.”—Health worker, Southern Province*

Some participants at management level noted that capacity that was built through ECHO and peer learning significantly contributed to personnel development in health facilities. As personnel in health facilities learned how other facilities handle difficult cases, they felt motivated and empowered to become confident in handling difficult cases that previously they would not treat.

*“One scenario that we had was a patient who had a viral load which was not coming down, it was just going up.… I recalled there was a discussion concerning that and they talked about different things that we could do to help our client if he grows up, and at the moment I have seen that things have actually worked well for that client and the viral load has actually come down.”—Manager, Southern Province*

Another manager noted that health facilities that participated in ECHO increasingly started using equipment that they had not been using because they did not have the capacity. Sometimes facilities were not using certain laboratory equipment because they had not encountered cases that required such equipment, and in other facilities, ECHO showed the need for orientation on how to use certain equipment.

##### Facility management support

Facility culture was also identified as a major enabler in ECHO implementation in some facilities, which enabled participation. In these facilities, implementation was helped by broad ownership of the program among staff at all levels.

*“…some of them we never even used to know their names. I just knew they existed somewhere and we would fight each other when they come to the ART department. But here we are even having lessons, presentations, training over the weekends and we are all just working as one.”—Manager, Lusaka Province*

Hence, the ECHO culture helped to bring everybody together, reduced over reliance on ECHO coordinators, and made it a team effort to remind one another on sessions but also made it everybody’s business to ensure that the link was shared. Thus, building an ECHO culture at facility level helped make staff at all levels ECHO champions. Further, it helped push for its implementation leading to improved ECHO implementation.

Facility leadership was also instrumental in ensuring that multiple mediums of communications were used in sharing of the link, such as use of memorandum, posting reminders of session on institutional notice board, sharing of the link using multiple social media platforms, such as WhatsApp. Further, because of strong facility leadership and ECHO culture, other cadres including students were involved in ECHO sessions. Because of good leadership, ECHO has been incorporated not just as a part of the curriculum for student but also as part of student assessments.

In some facilities, active leadership made it a point that those who missed sessions, benefit by participating in symposium and clinical meetings organized at the facility. In addition, facility leadership also tracked those who attended and ensured that the log books were filled in after each session. This enhanced commitment among key stakeholders and in turn helped ECHO implementation. Hub-level leadership was also cited as another key enabler. Participants shared that the practice of hub leadership sharing topics and schedules in advance helped facilitate ECHO implementation.

### Barriers intrinsic to ECHO approach

#### Centralization

Some respondents were of the view that the centralized system of coming up with topics was a demotivation as some of the issues that were tabled in ECHO sessions did not respond to or answer the critical challenges that providers and facilities were encountering. Other issues were time management, short notice to prepare ECHO cases, need for guidance for foreign presenters on the approved Zambian guidelines in HIV/TB, and how to access recorded sessions.

*You find that the management that is being discussed is not in the Zambian guidelines. So, you find that they are saying give this drug, but the guidelines are saying give this other drug. So, maybe it should be presented in such a way that it suits our country.—*Manager, Eastern Province

*In situations where—maybe in a discussion situation—where you had different views with what the very highest person you have has presented, you start to think, can I argue with my boss or can I dispute the consultant?—*Manager, Southern Province.

#### Perception of donor-driven programs

While some participants noted ambiguity in ownership and leadership of the initiative, sharing that when health workers perceive a project as donor-driven, rather than nationally owned, they do not feel compelled to participate.

When you look at the donors and the partners, the way they decided to introduce the program, they have put it in a way such that the support staff from the partners are the ones that are made to lead these sessions. It would have been better if they had penetrated through the facility management so that it becomes more of a government-driven program than a donor-driven program. So, once the staff [donors] leaves there will be no one to spearhead the program.—Manager, Lusaka Province

- Infrastructure requirements—The vast majority of respondents reported that internet connection in their area is poor and particularly challenging for rural health facilities. Related to this, power outages were cited as barrier to ECHO implementation. It was reported that load shedding had brought about intermittent power supply that ultimately affected ECHO session attendance. For example, at the time of this study, Chipata in Eastern Province experienced power outages on a Monday.
  Load shedding [is an] issue whereby it is time for ECHO and you do not have power, meaning you will not attend—Health worker, Chipata
- Program and context-specific barriers—Many program and context-specific barriers mirrored the enablers identified, reflecting the importance of tailoring capacity-building materials to diverse user groups and the influence of facility-level leadership and working environments on program implementation.

Alongside positive feedback on content, structure, and delivery of presentations reported above, some participants felt that further efforts are needed to make ECHO session more inclusive. First, although the engagement of various cadres and departments in ECHO sessions was highlighted as a factor motivating participation, some respondents noted that key cadres involved in HIV/TB services were not included, namely HIV testing counsellors, community-based volunteers, and peer educators.

I have noticed there is just one profession which does the presentations, we can have social workers… maybe a nutritionist.”—Heath Worker, Southern Province

In fact, many focus group discussion participants agreed that the topics were too medical (biased towards one cadre: doctors) thus did not reflect the multidisciplinary approach of medical care.

We are being left out more especially where nursing care is concerned. In a clinical setup, you cannot do away with nursing care because us nurses, we are always with the patient, giving them medication and giving them psychological care until they are discharged.—Manager, Western Province

I think we should increase more presentations on children, adolescents, and HIV pregnant women because in Zambia, management of pediatric ART is still a challenge.—Health worker, Southern Province

Related to this, many felt that the language used in presentation was too technical and too academic for some cadres, even those with prior training.

When a presentation has a lot of jargons … the discussion goes too much scientific. Therefore, you find that the discussion is only for those at that level, the other cadres are out of the discussion.—Health Worker, Western Province

When the one who is presenting is specialized in a particular topic, they are inclined to use the jargons within their specialty, forgetting they are dealing with a mixed group.—Health worker, Lusaka Province

Sometimes they are just too academic. I know they are quite detailed for clinical staff but bear in mind that in these hospitals, it’s not everyone maybe who did biochemistry—Health worker, Southern Province.

Opinions of presenter ability were wide ranging. Some respondents reported that some presenters lacked confidence, displayed lack of expertise in the assigned topics thus resorted to reading slides rather than engaging the audience. There was general concern that the overuse of technical jargon, poor audibility, being judgmental, or ridiculing or belittling participants, especially when providing feedback to case presentations, was a reason why some participants were uncomfortable volunteering to present cases or even participate in ECHO sessions.

Some participants reported that they felt more comfortable speaking and participating in local ECHO (provincial ECHO) than country-level ECHO. Further analysis of the data revealed individual attributes, such as the seniority (big titles) of some presenters during country-level ECHO, as barriers that prevented others who felt inferior from participating. Individual characteristics such as fear of making a mistake (“I will embarrass myself if I say something wrong and the whole country will know”).

As noted above, facility leadership and support were seen as critical components to successful implementation. However, while some participants highlighted facility leadership as a factor encouraging participation, others reported that disinterest in ECHO among facility managers affected participation. In Eastern Province for example, it was heard that some managers had a tendency of scheduling meetings during ECHO time and in some instances, these meeting were scheduled in venues earmarked for ECHO, thus sending a message that ECHO was not a priority.

Some respondents felt that facility support for ECHO was nonexistent, thus resulting in lack of enforcement in ensuring that health workers attended ECHO.

It’s a bit challenging for me as a departmental manager to convince everyone to come for the meeting, but if It comes from the facility manager, I think it will carry more weight and I really wanted my manager to be here, he is not around… they feel it’s for ART.—Manager, Eastern Province

Limited infrastructure at facility level was also cited as hindrance to ECHO participation. In cases where conference facilities were available, the venues were sometimes too small to accommodate the numbers. Infrastructure impacted on attendance greatly.

Finally, timing of ECHO sessions was flagged by some focus group participants as a barrier to implementation and an example of where intervention design could be less centralized and more end-user friendly. Specifically, participants were mixed in their views about Monday as an ECHO day—some, especially managers, expressed appreciation and appealed that the day (Monday) be maintained as it was a good day to have ECHO sessions. Considering that Monday is the first day of the week, it accorded them a chance to implement what is learned in the ECHO session. However, most of the respondents felt that that Monday was not a good day for ECHO sessions on account that Monday is a busy day.

The major drawback is on the days when the ECHO session is actually being held, because you cannot be in the ECHO session and again attend to the clients, so you find that the will be tasks shifting on that particular day, in the end not everyone will actually attend the ECHO session.—Manager, Eastern Province

Those against Monday shared that most of the critical services are unavailable over the weekend in most facilities hence most cases are pushed to Monday. In addition, some respondents reported that Monday was also a day earmarked for meetings in most facilities, making it less conducive for ECHO. General workload and time constraints were also highlighted as a barrier to ECHO participation. Patient numbers, shift change, and limited workforce were all issues highlighted by medical practitioners as working against them participating in ECHO sessions.

## Study limitations

Despite the positive contributions highlighted in this study, the study had limitations in that improvements in the outcomes of interest to implementation of the ECHO program at health facilities were subjected to other interventions supported by the Zambia MOH and other implementing partners working on HIV and TB programs.

## Discussion

The findings from this study indicate that implementation of ECHO in Zambia was followed by improvements in selected key performance indicators, namely TPT completions and improved viral load coverage. There was generally, an improvement in practices among HCW’s involved in the management of HIV/TB in facilities accessing ECHO. The ECHO intervention in Zambia has contributed to accelerating progress towards epidemic control given that viral load testing is a gold standard for monitoring processes towards achieving reduced transmission and managing HIV disease.

The observed improvements in selected patient HIV-related outcomes was because of adherence to subject matter expert recommendations on the management of HIV/TB cases to HCWs (health care workers) accessing ECHO sessions. These findings are similar to findings from the study conducted in Namibia where ECHO project sessions resulted in the significant increase in health workers accessing ECHO sessions [12]. However, it is important to acknowledge that there are multiple factors that impact on HCWs practices such as lack of supplies and diagnostic equipment which were observed this study.

Despite showing that there was improvement in outcome indicators in both ECHO and non-ECHO implementing facilities, further statistical analysis showed that the improvements in TPT completions and viral load coverage were statistically significant in ECHO implementing facilities. These findings are similar to findings from many studies [14, 15] where ECHO implementing facilities showed significant improvements in TPT completions and viral load coverage as well as strengthened capacity of the HIV workforce [14].

The strength of this study lies in the demonstration of impact of ECHO model at patient-level health outcome. The investigators however, acknowledge that this study had limitations in that, other interventions such as HIV/TB clinical mentorship program implemented alongside ECHO and HIV care and service delivery support from various stakeholders could have influenced these outcomes.

Consequently, as Zambia strives to achieve the 95 95 95 UNIADS targets and attain HIV epidemic control by 2030, the ECHO program role in the process becomes pivotal to the successful attainment of these goals. It therefore becomes imperative to highlight the impact and uptake of ECHO on HIV/TB services to get the full support of policy makers, funding agencies, implementing partners and all stakeholders involved in the provision of HIV/TB services. The multilayered support will be necessary in the ongoing country wide establishment of the HIV/TB ECHO program in health facilities. Notwithstanding the challenges of funding from central level to support this high-impact intervention, there is need for all health facilities to invest in resource allocation for continued support to the ECHO model for maintenance to achieve sustainability of the program.

## Data Availability

The datasets during and/or analyzed during the current study are available from the corresponding author on reasonable request.
